# Management of Lateral Epicondylitis: A Narrative Literature Review

**DOI:** 10.1155/2020/6965381

**Published:** 2020-05-05

**Authors:** Kun-Long Ma, Hai-Qiang Wang

**Affiliations:** ^1^Department of Orthopedics, Yongchuan Hospital of Chongqing Medical University, Hua Road, No. 439, Yongchuan, Chongqing 402160, China; ^2^Institute of Integrative Medicine, Shaanxi University of Chinese Medicine, Xixian Avenue, Xixian District, Xi'an 712046, Shaanxi Province, China

## Abstract

Lateral epicondylitis, also termed as “tennis elbow,” is the most common cause of elbow pain and dysfunction, mainly resulting from repetitive gripping or wrist extension during various activities. The exact pathogenesis remains largely elusive with putative tendinosis, a symptomatic degenerative process of the local tendon. It is usually diagnosed by clinical examinations. Sometimes, additional imaging is required for a specific differential diagnosis. Although most cases can be self-healing, the optimal treatment strategy for chronic lateral epicondylitis remains controversial. This article presents a landscape of emerging evidence on lateral epicondylitis and focuses on the pathogenesis, diagnosis, and management, shedding light on the understandings and treatment for healthcare professionals.

## 1. Introduction

Lateral epicondylitis (LE) was first described in the English literature by Runge in 1873 [1]. It was described as chronic symptomatic degeneration of the forearm common extensor tendon attachment at the humeral ectocondyle. It is one of the most common overuse syndromes in primary medical care. LE affects 1% to 3% of the population, mainly those middle-aged people without gender difference [2]. LE can produce a great social and economic burden due to lost workdays and can even disable some patients from working for weeks [3, 4]. Despite advances in the treatment of LE, there is still a lack of established standards. It is generally self-limiting, and most cases require no treatment, with up to 80% cases recovering within one year [5]. Patients with refractory symptoms may require further conservative or surgical treatments.

## 2. Pathogenesis

The exact etiology of LE has not been well identified. However, it is commonly associated with repetitive microtrauma from excessive gripping or wrist extension, radial deviation, and/or forearm supination [6, 7]. The extensor carpi radialis brevis (ECRB) is the most frequently affected muscle. The pronator and other extensor carpal muscles are also commonly affected [8]. In addition to the factor of excessive mechanical forces, the unique origin of ECRB in the lateral aspect of the capitellum places the tendon at risk for repeated undersurface abrasion during elbow extension and flexion [9]. LE was originally considered as an inflammatory process, especially in its initial phases. Repetitive microtrauma resulting from overload or overuse can cause collagen fibril rupture and the activation of the innate immune system [10, 11]. However, histopathological studies have shown that there is absence of inflammatory cells in biopsies of chronic LE [12, 13]. Accumulating evidence identifies it as tendinosis, a symptomatic degenerative process characterized by an abundance of fibroblasts, vascular hyperplasia, and unstructured collagen. These findings were termed as angiofibroblastic hyperplasia by Nirschl and Alvarado [14]. The mechanical properties of tendons are commonly determined by the structure of protein molecular and the composition of the extracellular matrix [15]. Strain upon a tendon normally promotes cross-linkage and collagen deposition [13]. In situations of repetitive stretching, multiple microtears of the tendon potentially cause an irreversible denaturing of matrix proteins and proliferation of fibrous tissue [16]. Over time, these scar tissues are vulnerable to repetitive forces, with subsequent further tears. High-frequency cyclical trauma and immature repair result in more severe tears, with consequent alteration and failure of musculotendinous biomechanics and worsening of symptoms [17].

Emerging evidence indicates a significant link between the strain degree of tendons and the extent of injuries [18, 19]. Strains less than 4% generally allow the tendon restore its original length after unloading, but the collagen fibers begin to fail when the strains are more than 4%, and it will be prone to rupture when the strains are over 8%. Kraushaar and Nirschl [13] described four stages of tendinosis, facilitating the recognition of the degenerative process of LE (Table 1). Notwithstanding the main cause is degeneration, additional pathophysiological mechanisms also contribute to the development of tendinosis. LE patients with painful symptoms often involuntarily lead to “underuse” or stress shielding of affected tendons, which subsequently results in structural weakening of the tendon, making it more sensitive to injury [18]. Meanwhile, increasing shear forces promotes fibrocartilaginous formation at tendon enthesis, which contributes to weakening at the tendon-bone junction and initiating development of tendinosis [20].

Histopathological studies have shown defects and necrosis inside the tendon fibers within tendons in patients with chronic LE, which is ascribed to strong association with underuse of the affected limb due to pain-related immobilization [20]. In addition, inadequate tendon angiogenesis and continuous muscle contraction can lead to tendon ischaemia, which further aggravates the development of tendinosis [21].

As for the pain machinery of LE, most studies ascribe the pathogenesis of LE to neurogenic etiology based on several lines of evidence indicating the presence of nerve fibers with reactivity to neuropeptides, including substance P (SP) and calcitonin gene-related peptide (CGRP) [22–24]. Ljung et al. [22] observed 5 patients with LE and 4 patients with medial epicondylitis (ME) by immunohistochemistry, indicating that the SP/CGRP innervation was present in the pathologic tendon tissues of LE as well as ME patients. Neurokinin-1 receptor immunoreaction was noted as varicose fibers in the form of a single fiber or nerve bundles. Thus, the findings present emerging evidence for a possible neurogenic pathogenesis of LE and ME. Uchio et al. [23] concluded that neuropeptides (SP and CGRP) and cytokines (interleukin-1*α* (IL-1*α*) and tumor growth factor-*β* (TGF-*β*)) might be involved in the pathogenesis of LE. However, further studies are needed to clarify the intrinsic relationship between neuropeptides and cytokines. Furthermore, Han et al. [24] studied the mRNA levels of neuropeptides and cytokines in LE with corticosteroid injection treatment. *In vivo* study found that the expression of SP mRNA was maximally inhibited by corticosteroid triamcinolone acetonide (TAA) at 24 hours but recovered at 72 hours. CGRP mRNA and IL-1*α* mRNA were inhibited at 24 and 3 hours, respectively. Consequently, the reaction mechanism of the corticosteroid for relieving pain in LE is mainly achieved by inhibiting the expression of neuropeptides and cytokines. Besides, a significant positive correlation between CGRP and IL-1*α* was also noted after 72 hours of TAA treatment, implicating the role of neurogenic inflammation in the pathogenesis of LE.

## 3. Clinical Evaluation

Patients often complain of pain or burning around the lateral epicondyle of the humerus, which frequently radiates down the forearm and sometimes extends proximally to the upper arm. This pain is usually triggered or exacerbated by a variety of activities involving wrist extension under resistance, such as grasping objects or twisting towels [25, 26]. The degree of the pain often ranges from mild to severe degrees and from intermittent to persistent, which seriously affects patients' daily life quality. In addition, patients often complain of weakness on gripping and difficulty in lifting [27]. During physical examinations, marked tenderness is usually inspected at the origin of the ECRB in the lateral epicondyle [28]. The pain can be exacerbated with resisted wrist extension, middle finger extension, and forearm supination with the elbow in the extended position. Usually, normal elbow motion can be preserved even in some severe cases [26].

Nirschl and Ashman [29] proposed a classification system and thus separated LE into seven phases based on the level of pain (Table 2). Although there is no complete correlation between histological lesions and clinical features of each phase, their supposed theoretical correlation is helpful to guide the treatment of LE.

## 4. Diagnosis

Most cases of LE can be clinically confirmed by thorough history inquiry and physical examinations. The contents of medical history collection usually include occupation, hand dominance, daily behaviors and habits, duration of symptoms, date of prior episodes, number of recurrences, inducing or aggravating factors, treatment modalities, and tobacco use. The duration of symptoms and number of recurrences are two key important factors to determine the stage of LE [30].

Any test capable of triggering the typical symptoms of LE can be considered as an effective examination modality for diagnosing LE. Resistance of the middle finger extensor can cause elbow pain due to selective recruitment of the ECRB tendon [31]. Resistance of wrist extensors with full elbow extension and pronation can reproduce the pain in mild-to-moderate cases [25]. Special tests are commonly used during the physical examination, such as the chair test, Cozen's test, and Mill's test [32, 33]. Chair test requires the patient to lift a chair with the shoulder adducted, elbows extended, and forearms pronated. Pain on the lateral epicondyle indicates lateral epicondylitis. Cozen's test requires the patient seated, with the elbow extended, forearm maximal pronation, the wrist radially abducted, and the hand in a fist. Then, the examiner moves the wrist to dorsal flexion and moves the wrist towards palmar flexion. Mill's test requires the patient seated, elbow extended, and forearm pronated. Then, the examiner moves the wrist passively in palmar flexion and hereby stretching the extensors.

Besides, grip weakness is also been considered as an effective test, with 83% accuracy in determining LE [27]. However, when clinical symptoms cannot be well defined based on physical examination and history, diagnostic imaging may be needed. Although negative findings are usually noted for radiographs, useful information can be obtained in terms of revealing bone diseases, such as arthropathy, osteochondral defects, loose bodies, and calcifications of ECRB origin [26]. Although CT is more sensitive than MRI in identifying tears of capsule, it is rarely used in the diagnosis of LE because of ionizing radiation [34].

Ultrasound is considered as an efficient, noninvasive, and relatively cost-effective imaging method for LE [35]. There are a variety of findings on ultrasound for identifying degenerative changes of the tendons attached to the region of the lateral epicondyle, which includes bone irregularities, calcific deposit, thickening, thinning, and tears of affected tendons or capsule [36]. Moreover, neovascularization can also be detected by ultrasound. If none of these findings is detected, LE can be probably ruled out [37].

In comparison with ultrasound, MRI can provide a better view of the complete anatomical structures of the lateral epicondyle [38]. Primary findings of elbow MRI include signs of abnormal thickening tendon and capsule and increased signal intensity within the common extensor origin. MRI can also identify partial or full-thickness tears of the ECRB, which can influence the need for surgical management and be helpful during preoperative planning [39]. In comparison with ultrasound, however, MRI is of limited diagnostic value in determining the overall extent and size of tendon tearing [40]. MRI is usually considered for the possible intra-articular pathology. It is not recommended routinely owing to its cost and the inconsistence of clinical symptoms with imaging findings [41].

LE is the leading cause of elbow pain; however, similar pain caused by other diseases should be carefully identified to avoid misdiagnosis. These potential diseases mainly include cervical radiculopathy, frozen shoulder, radial tunnel syndrome, lateral plica syndrome of the elbow, posterolateral elbow instability, and inflammatory edema of the elbow muscle. Other causes of pain include low-grade infection or other inflammatory diseases, such as rheumatoid arthritis.

## 5. Treatment

A variety of treatment options have been recommended for LE. Unfortunately, there are still no universally accepted therapeutic modalities. However, the treatment of LE usually has five therapeutic goals: controlling elbow pain, preserving movement of the affected limb, improving grip strength and endurance, restoring normal function of the affected limb, and preventing further deterioration [26]. Nonoperative treatment remains the priority and mainstay for most patients with LE. Surgical intervention is available for recalcitrant cases.

### 5.1. Nonoperative Treatment

Nonoperative treatment can significantly resolve the symptomatic LE in 90% of cases [42, 43]. Nonoperative care usually includes activity modification, physiotherapy, nonsteroidal anti-inflammatory medications, bracing, extracorporeal shock-wave therapy, and acupuncture. With a promising result, biotherapy method has been very popular in recent years, including autologous blood injections (ABI) and platelet-rich plasma injections (PRP).

#### 5.1.1. Activity Modification

Modification of activity and avoidance of overwork are essential components for any treatment protocol. Turning the palm up while lifting and avoiding palm-down exercises can transfer the force away from the lateral epicondyle to the medial epicondyle and help alleviate lateral elbow pain. Besides, LE patients should be advised to correct adverse living habits and stay away from some inciting activities. The principle of RICE (rest, ice, compression, and elevation) can be helpful to relieve pain at the initial stage.

#### 5.1.2. Physiotherapy

Various physiotherapy modalities are recommended for the treatment of LE. Traditional treatment options include electrotherapeutic and nonelectrotherapeutic modalities, aiming for improving function and reducing pain by stretching and strengthening the affected wrist extensors [44–48]. Recently, eccentric exercise (EE) has gradually been a first-line conservative treatment for LE. EE is executed via stretching the musculotendinous unit with an applied load [49]. Clinical trials have demonstrated that the EE has superior efficacy in the treatment of LE, in comparison with therapeutic ultrasound, [50] bracing, [51], and a combination of multiple interventions [52]. Although the EE has a promising outcome, the exact mechanisms underlying EE in treating LE remain ambiguous due to varied eccentric programs and undefined optimal dosing [53].

#### 5.1.3. Anti-Inflammatory Medications

Five recent placebo-controlled trials demonstrate that topical nonsteroidal anti-inflammatory medications are effective within four weeks in the treatment of LE [54–58]. There have been no consensuses on the superiority of oral versus topical NSAIDs in pain control, though oral NSAIDs may cause gastrointestinal adverse effects [59]. Hay and colleagues reported that corticosteroid injection was superior than NSAIDs in improving patients' outcomes within four weeks, without long-term benefits at 12 months [43]. Other studies also found that despite of its short-term pain relief, corticosteroid injection is inferior than watchful waiting or physical therapy at one year follow-up [60, 61]. Notably, repeated injections of the corticosteroid may result in iatrogenic tendon rupture and muscle atrophy. Therefore, clinicians should be alert to the abuse of corticosteroids in the treatment of LE on account of poor long-term efficacy and potential adverse effects [62].

#### 5.1.4. Counterforce Braces

Counterforce bracing has been popular in the treatment of LE for decades. Using counterforce braces can significantly alleviate pain by pressing on the forearm extensor muscles and then inhibiting and dispersing the stress on the origin of affected ECRB, thereby facilitating its self-repair [29]. Biomechanical studies have shown that immobilizing the forearm with braces can significantly lessen the stress on the ECRB origin [63]. The latest randomized controlled double-blind trial shows that the use of counterforce brace can significantly decrease the frequency and severity of pain for 2–12 weeks and improve the elbow function at 26 weeks, compared with the placebo group [64]. In addition to counterforce braces, cock-up wrist braces during activities of daily living can limit wrist extension and firing of the ECRB tendon, allowing the injured tendon to heal [65].

#### 5.1.5. Extracorporeal Shock-Wave Therapy

Extracorporeal shock-wave therapy (ESWT) is one of the commonly used physical therapy modalities for treating LE, in spite of conflicting results in the available literature. The mechanism of ESWT has not been completely clarified, possibly including direct stimulation of healing, neovascularization, direct suppressive effects on nociceptors, and a hyperstimulation mechanism blocking the gate control [66]. ESWT may not reverse the pathology of LE but improve the symptoms of LE. ESWT is not appropriate for acute LE but is recommended when symptoms persist for more than 6 months or when other conservative treatments fail [67].

#### 5.1.6. Acupuncture

Acupuncture is a green, simple, inexpensive, and beneficial treatment for musculoskeletal diseases, especially for dysfunction and pain symptoms [68]. However, current data from evidence-based medicine indicate conflicting results. Two systematic reviews have not concluded whether acupuncture was effective for LE [69, 70], whereas three systematic reviews suggest that acupuncture is very effective in relieving LE pain in the short term, with the long-term results remaining unclear [71–73].

#### 5.1.7. Autologous Blood Injection (ABI)

Local ABI has been proved effective and widely used for treatment of LE. There are two hypotheses for the mechanism of ABI. On the one hand, ABI works by initiating the inflammatory response around the affected tendon, which may result in cellular and humoral mediators to induce a healing cascade [74]. On the other hand, ABI allows delivery of growth factors inducing fibroblastic mitosis, triggering stem cells, and angiogenesis, probably promoting angiogenesis and collagen formation [75]. Current evidence suggests that ABI can achieve good outcome in the short term; however, no benefit has been found in the medium- or long-term follow-up [76, 77]. In addition, it should be noted that ABI has high risks of injection site pain and skin reaction. Accordingly, its indications should restrict to those recalcitrant cases when other modalities of treatment are less effective.

#### 5.1.8. Platelet-Rich Plasma (PRP) Injection

PRP has gained popularity in recent years in the treatment for LE. The exact mechanisms of PRP remain unknown. There are theories attributed to platelets releasing high concentrations of platelet-derived growth factors enhancing wound healing, bone healing, and tendon healing [78]. However, available studies have reported conflicting results, which make it difficult to draw clear conclusions on PRP for LE. The latest systematic review manifested that PRP injection has no obvious effects on the treatment of chronic LE [79]. Several studies have shown that PRP does not provide significant benefits over corticosteroids, ABI, or even saline injections [80–82], whereas other studies reported better results with pain relief and function improvement [83, 84].

### 5.2. Operative Treatment

Surgical intervention can be an option for patients with persistent pain and disability that have failed appropriate nonoperative management.

The number of patients requiring surgical treatment is estimated about 4% to 11% [85]. There are mainly three surgical approaches, i.e., open, percutaneous, and arthroscopic techniques. The surgical focus is to debride the degenerated portion of the ECRB with or without repairing the ECRB tendon [86, 87]. Evidence in the literature indicates fair to good results for these procedures, presenting surgeons with many options for treatment. However, there have been no definite understandings for the mechanism of good outcome.

#### 5.2.1. Open Surgery

Open surgery involves a small lateral incision with dissection and degenerated tendon identification. After debridement of denatured tendon tissues, the main structure of the tendon can be repaired, lengthened, and fixed by drilling or decortication of the lateral epicondyle [88, 89]. Nirschl and Pettrone [90] reported 88 elbow surgical cases out of clinical series of 1,213 patients which involved excision and repair of the ECRB tendinosis tissue. The short-term outcomes of the original procedure were described as good to excellent by 85% of patients with an overall improvement rate of 98% and a return to full activity in 85% of patients [90]. In a recent retrospective study, Dunn et al. [91] presented 10- to 14-year follow-up results of the Nirschl surgical technique for 83 LE patients with 92 elbows. Eighty-four percent of elbows were reported little or no pain, and 92% patients returned to normal elbow range of motion, while 93% of patients could return to their sports. The overall improvement rate was 97%.

Coleman et al. [92] reported their 15 years of experience in treating refractory LE. Amongst 158 consecutive patients treated with open surgery, 94.6% achieved good or excellent results at an average follow-up of 9.8 years. Although the results of open surgery are positive, there is also a risk of instability of the elbow since excessive dissection of the LE may injure the lateral ligaments.

#### 5.2.2. Percutaneous Surgery

Percutaneous surgical approach is mainly used for releasing the common extensor tendon origin at the lateral epicondyle. This technique has been demonstrated to be safe, reliable, and cost-effective [93, 94]. Good midterm outcomes in pain relief have been widely reported with a percutaneous surgical approach [95,96]. However, Pierce et al. [97] reported that arthroscopic and open techniques achieved a better prognosis than the percutaneous surgical approach for the treatment of LE.

In recent years, a novel technique termed as ultrasound-guided percutaneous tenotomy (UGPT) has been reported as a safe and effective procedure for the treatment of LE, with durable improvements in terms of symptoms, function, and ultrasound imaging at 1-year follow-up [98]. Barnes et al. [99] reported similar outcomes for 19 patients with chronic, refractory lateral, or medial elbow tendinopathy up to 1 year after the procedure. This novel procedure requires the assistance of the TX1 Tissue Removal System (Tenex Health, Lake Forest, CA), which is performed through an approximately 5 mm incision and uses ultrasonic energy to remove diseased tendon tissue in the damaged region, creating an acute inflammatory reaction and facilitating tendon healing [100].

Seng et al. [101] reported 20 patients with refractory LE treated with UGPT through TX1 Tissue Removal System. The results demonstrated that UGPT procedures could provide sustained pain relief and functional improvement for recalcitrant cases at 3-year follow-up.

Boden et al. [102] compared the effects of PRP and UGPT procedures in the treatment of medial and LE. No statistically significant difference was found between the two treatment modalities. They concluded that PRP and UGPT procedures were both effective in aspect of pain relief and the improvement of function and life quality.

#### 5.2.3. Arthroscopic Surgery

Elbow arthroscopy has been used for the treatment of LE as well. It was first described by Baker and considered as a minimally invasive and efficient surgical procedure [103]. The major advantages of this procedure are quick return to work and the ability to treat the potential intra-articular pathology through visualization of the entire elbow joint. Baker et al. [103] reported that 87% of LE patients undergoing elbow arthroscopy had good long-term follow-up results. Various studies have shown a lower complication rate of arthroscopic treatment than that of open and percutaneous approach [104–106]. However, recent systematic review studies reported a compromise result, demonstrating no differences among open, arthroscopic, and percutaneous surgical techniques for LE regarding the duration of return to work, complication rate, or patient satisfaction [97, 106]. Although there are generally positive results, elbow arthroscopy is thought to have a demanding learning curve with potentially risks of damage to the radial nerve and the lateral ulnar collateral ligament [107–109].

## 6. Conclusions

LE is a common cause of pain and disability affecting patients aged between 35 and 55 years. Most cases have a self-limiting course of between 12 and 18 months. However, symptoms can be persistent and refractory, thus needing interventional measures. Nonoperative treatment remains the priority and mainstay for LE. Most cases can be well treated with multiple nonoperative treatments, with as high as 90% success rate. However, there is no evidence suggesting the superiority of nonoperative treatment options. When nonoperative treatment fails, three surgical interventions will be recommended for patients with lateral LE, including open, percutaneous, and arthroscopic approaches. Similarly, no conclusions on the effectiveness of surgical interventions can be reached mainly due to a lack of high-quality evidence and inconsistent outcome measures.

## Figures and Tables

**Table 1:** Pathologic stages of lateral epicondylitis.

Stage	Degenerative changes of tendinosis
I	Peritendinous inflammation with no pathological alterations
II	Involving pathological alterations such as tendinosis or angiofibroblastic degeneration
III	Involving pathological changes and complete structural failure
IV	Involving fibrosis, soft matrix calcification, and hard osseous calcification, in addition to the features of stage II or III

**Table 2:** Clinical classification of lateral epicondylitis phases.

Phase	Description of pain changes of different phases
I	Mild pain after activity, usually recovers within 24 hours
II	Mild pain more than 48 hours after activity, no pain during activity, can be relieved with warm-up exercises, and recovers within 72 hours
III	Mild pain before and during activity, no significant negative impact on the activities, and can be partially relieved with warm-up exercises
IV	Mild pain accompanies the activities of daily living and has negative impact on the performance of activities
V	Harmful pain unrelated to activities, great negative impact on the performance of activities but does not prevent the activities of daily life. Need complete rest to control the pain
VI	Persistent pain despite complete rest and can prevent the activities of daily life
VII	Consistent pain at rest, aggravated after activities, and disturbed sleep

Notes: the pain in phases I and II is usually self-limiting with due care and protection; the pain in phases III and IV usually needs some nonoperative treatments; and the pain in phases V–VII is more likely to require operative treatment.

## Data Availability

The updated article data in the literature used to support the findings of this study are from previously reported studies and datasets, which have been cited. The processed data are listed as Table 1 and Table 2.
